# Is ghrelin a biomarker of early-onset scoliosis in children with Prader–Willi syndrome?

**DOI:** 10.1186/s13023-021-01930-1

**Published:** 2021-07-08

**Authors:** Dibia Liz Pacoricona Alfaro, Gwenaelle Diene, Graziella Pinto, Jean-Pierre Salles, Isabelle Gennero, Sandy Faye, Catherine Molinas, Marion Valette, Catherine Arnaud, Maithé Tauber

**Affiliations:** 1 CERPOP, Toulouse University, Inserm - Paul Sabatier University, Toulouse, France; 2 Endocrinology, Obesity, Bone Diseases, Genetics and Gynecology Unit, Children’s Hospital, University Hospital Center of Toulouse, Toulouse, France; 3 French National Reference Center for Prader-Willi Syndrome, Children’s Hospital, University Hospital Center of Toulouse, 330, Avenue de Grande-Bretagne – TSA, 70034 - 31059 Toulouse Cedex 9, France; 4 Pediatric Endocrinology, Diabetology and Gynecology Department, Assistance-Publique Hôpitaux de Paris (AP-HP), Necker-Enfants Malades Hospital, Paris, France; 5 Toulouse Institute of Infectious and Inflammatory Diseases (Infinity), Inserm UMR1291 - CNRS UMR5051, University Toulouse III, Toulouse, France; 6 French National Reference Center for Rare Diseases of Calcium and Phosphate Metabolism - ERN BOND, University Hospital Center of Toulouse, Toulouse, France; 7 Clinical Biochemistry and Molecular Biology Department, Federative Institute of Biology, University Hospital Center of Toulouse, Toulouse, France; 8 Pediatric Axis of the Clinical Investigation Center (CIC) 9302/Inserm, Children’s Hospital, University Hospital Center of Toulouse, Toulouse, France; 9 Clinical Epidemiology Unit, University Hospital Center of Toulouse, Toulouse, France

**Keywords:** Prader–Willi syndrome, Early-onset scoliosis, Total ghrelin, Acylated ghrelin, Unacylated ghrelin, AG/UAG ratio

## Abstract

**Background:**

Adolescents with idiopathic scoliosis display high ghrelin levels. As hyperghrelinemia is found in patients with PWS and early-onset scoliosis (EOS) is highly prevalent in these patients, our aims were to explore (1) whether ghrelin levels differ between those with and without EOS and correlate with scoliosis severity, and (2) whether ghrelin levels in the first year of life are associated with the later development of EOS.

**Methods:**

We used a case control study design for the first question and a longitudinal design for the second. Patients with PWS having plasma ghrelin measurements recorded between 2013 and 2018 in our database were selected and 30 children < 10 years old with EOS and 30 age- and BMI-matched controls without EOS were included. The Cobb angle at diagnosis was recorded. In addition, 37 infants with a ghrelin measurement in the first year of life were followed until 4 years of age and assessed for EOS. Total ghrelin (TG), acylated (AG) and unacylated ghrelin (UAG), and the AG/UAG ratio were analyzed.

**Results:**

EOS children had an AG/UAG ratio statistically significantly lower than controls. The Cobb angle was positively correlated with TG and UAG. TG and AG in the first year of life were higher in infants who later develop EOS without reaching a statistically significant difference.

**Conclusions:**

Our results suggest that ghrelin may play a role in the pathophysiology of EOS in PWS. Higher ghrelinemia in the first year of life required careful follow-up for EOS.

## Introduction

Ghrelin, a 28-amino acid peptide hormone, was first described in 1999 in its acylated form (AG). It is the endogenous ligand of the GHSR1a receptor [1]. Recent studies show that the non-acylated isoform (unacylated ghrelin—UAG) functionally inhibits AG and has activities independent of and synergistic with AG [2, 3]. Circulating total ghrelin (TG) levels showed peak in early life and then a progressive decrease with age and body mass index (BMI). The relationship between AG and UAG varies over time, and the AG/UAG may thus fluctuate across life stages and with pathological states [4, 5]. This multifaceted hormone has broader effects than appetite and metabolic regulation [6, 7]. Interestingly like other metabolic hormones, several in vivo and in vitro studies have demonstrated effects on bone metabolism and growth. Its effects on growth are exerted in part by the secretagogue effect of growth hormone (GH), but it also acts directly on the growth plate of long bones, stimulating chondrocyte proliferation and promoting endochondral growth [8].

Recent studies have documented increased ghrelin levels in adolescent girls with idiopathic scoliosis [9–11]. Scoliosis is a deformity in the three spinal axes with an angle on the frontal plane (Cobb angle) ≥ 10°. It most frequently affects peri-pubertal girls with a lean constitution and, in this case, is called adolescent idiopathic scoliosis (AIS) [12]. Scoliosis may appear before 10 years and is called early-onset scoliosis (EOS). Etiology of EOS is heterogeneous and can be classified into congenital, neuromuscular, syndromic or idiopathic [13].

In 2015, we showed that girls with AIS had ghrelin levels twice as high as controls [9]. In 2018, Yu et al. confirmed these results [10], and showed that ghrelin levels were higher in adolescent girls with progressive scoliosis than in those with stable scoliosis. Recently, the same team reported that ghrelin upregulated the expression of specific genes in the chondrocytes of AIS adolescents, which can lead to abnormal cartilage development [14]. In 2020, Xiao and colleagues showed dysregulation of the ghrelin/RANKL/OPG pathway in AIS adolescents, which can contribute to osteopenia [15]. Our recent study showed that, in vitro, ghrelin stimulates differentiation and mineralization of mature osteoblasts through the GHSR/GI/cAMP pathway. In the primary osteoblastic cells of patients with AIS a deregulation of this pathway was observed suggesting resistance to ghrelin in these patients [16].

Prader–Willi syndrome (PWS) is a rare genetic neurodevelopmental disease with an estimated prevalence at birth of 1/20,000 [17]. A specific nutritional, endocrine and neurodevelopmental trajectory is characteristic of this disease from severe hypotonia with poor suck and anorexia at birth to excessive weight gain preceding hyperphagia and obesity starting in early childhood [18]. Since 2002, several studies reported hyperghrelinemia starting early in life in patients with PWS [19–21] while circulating ghrelin is significantly lower in other causes of obesity compared to lean controls. We also showed that patients with PWS display high circulating ghrelin levels at all ages and that hyperghrelinemia precedes obesity [22–24]. The increased ghrelin values are not always due to the same ghrelin isoform. The different isoforms of ghrelin vary throughout life even though there is a wide range of variability from one person to another. Interestingly, we were able to show that the high plasma ghrelin levels in infants with PWS are due to a relative excess of UAG [23, 25] that may explain their poor appetite albeit displaying excessive fat mass *vs.* age- and BMI-matched controls. Conversely, we demonstrated that children and adult patients in the hyperphagia phase show higher AG levels with a relative deficit of UAG compared with controls of similar age and therefore, a AG/UAG ratio higher than in the general population [24]. We thus concluded that the evolution of the AG/UAG ratio is impaired in PWS *vs.* controls with a switch from an excessive UAG to an excessive increase of AG and a relative deficit of UAG levels starting occurring during the first 4 years of life [22–24] and this increased AG/UAG ratio remains afterwards during the hyperphagia phase of the disease.

In addition to eating and metabolic disorders, patients with PWS display many comorbidities, with a high prevalence of orthopedic problems including scoliosis, kyphosis and hip dysplasia. The prevalence of scoliosis is around 40% [26, 27], compared with 1–3% in the general population [28]. The scoliosis prevalence in PWS increases with age, from 23% in children under 4 to 75% in adults [29, 30], with both sexes equally affected [27]. The age of diagnosis has a bimodal distribution: the first peak in early childhood and the second in the pre-pubertal period. In childhood, EOS has mostly C-shaped curves and is comparable to the malformative scoliosis observed in other neurodevelopmental and polymalformative diseases, possibly related at least partly to hypotonia, whereas later scoliosis behaves similarly to AIS [30, 31] although these patients display increased BMI and/or fat mass.

Given that patients with PWS demonstrate elevated ghrelin levels and a higher prevalence of scoliosis, and that scoliosis is associated with high ghrelin levels, we hypothesize that hyperghrelinemia makes them vulnerable to scoliosis. To our knowledge, no study has explored this hypothesis. Our study focuses on EOS in PWS and investigates (i) whether ghrelin levels at diagnosis of scoliosis differ between PWS children with and without EOS, and correlate with scoliosis severity and (ii) whether ghrelin concentrations in the first year of life before GH treatment are associated with the later EOS development.

## Methods

### Study design and setting

This study is based on observational data from the French Reference Center for PWS (FRC-PWS), which collects data from 20 hospitals that follow children with PWS in France. The pediatric FRC-PWS database comprises patients with confirmed genetic diagnosis of PWS who are enrolled after parents or legal guardians give written consent. Sociodemographic information and health/medical data including comorbidities and treatments are collected by physicians in usual practice settings [32, 33]. The FRC-PWS database was approved by the French regulatory authorities. From 2013 to 2018, patients from the whole country who had been hospitalized at least once at the Toulouse University Children's Hospital or the Necker-Enfants Malades Hospital in Paris (pediatric sites for FRC-PWS) underwent plasma ghrelin measurement. The measurements were performed in the framework of clinical research projects (the European blood bank study, N°RCB: 2012-A01153-40, and the OXYJEUNE study, No EudraCT: 2016-003273-18).

This study used both matched case–control and longitudinal designs to examine our two hypotheses.

### Study participants

**Fig. 1:**
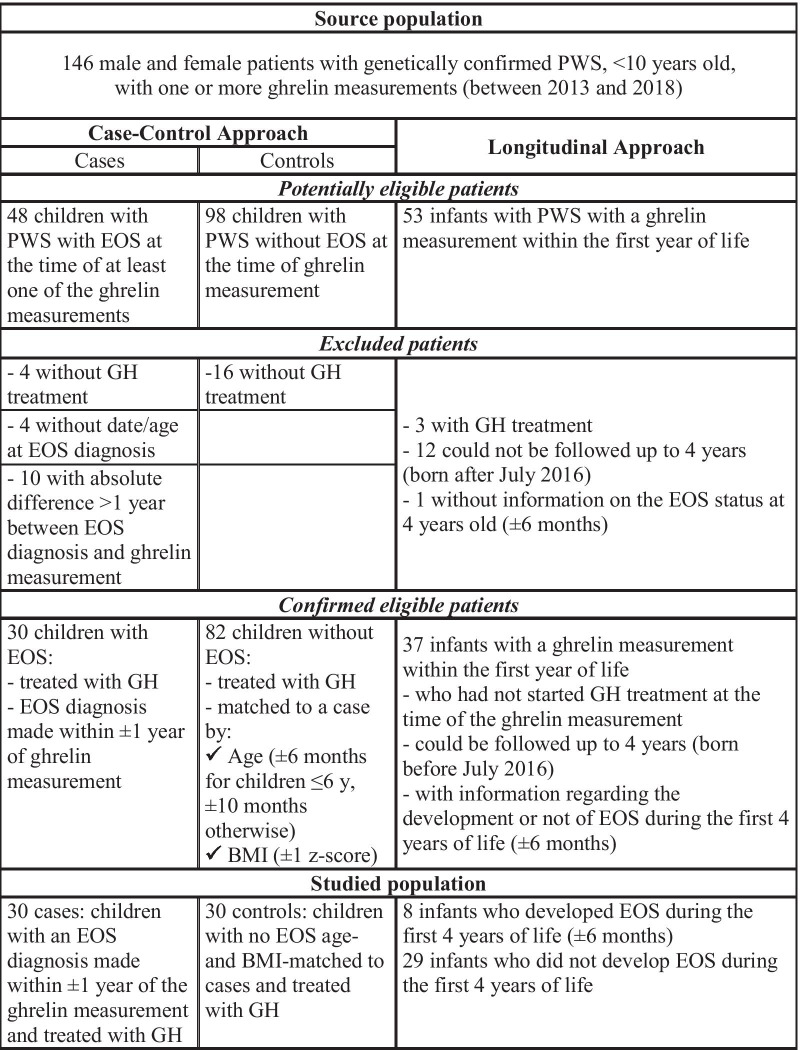
Flowchart of the study. *Note*: 14 patients were included in the study population of both approaches, of which 6 patients had scoliosis

Patients of both sexes < 10 years old with one or more ghrelin measurements were potentially eligible for this study (Fig. 1). The first group included 30 cases defined as children with PWS treated with GH, confirmed EOS diagnosis, and ghrelin levels measured at the time of scoliosis diagnosis (± 1 year). All the patients with PWS followed by the FRC-PWS are seen at least once a year by an orthopedist and underwent a spine X-rays to confirm or exclude the scoliosis diagnosis by Cobb angle. For three children whose scoliosis was diagnosed at other centers by the orthopedists who followed them, the value of the Cobb angle at diagnosis was not collected in the data base while there is no doubt on the diagnosis of scoliosis. The control group included children with PWS without EOS (excluded by X-ray) within ± 1 year of the ghrelin measurement and matched with cases for age (± 6 months for children ≤ 6 yrs, ± 10 months otherwise) and body mass index (BMI, ± 1 z-score). In one case–control pair there was a poor match on BMI (case = 4.5 z-score, control = 3 z-score) and age (25 months older for the control).

We also selected a sample of 38 infants with PWS with ghrelin measurement in the first year of life before the start of GH treatment. Birth dates ranged from November 2012 to June 2016. Patients’ records (including X-rays and orthopedist report) were examined to identify if they developed EOS after the ghrelin measurement and during the first 4 years of life. This information was obtained for 37 of them. Eight children developed EOS and the Cobb angle at diagnosis of scoliosis was obtained for 6 of them. The two patients with missing value were diagnosed at other centers by the orthopedists who followed them although precise data on the Cobb angle value was not collected.

### Sample collections for plasma ghrelin measurements

The ghrelin samples were taken after a 4-h fast and for infants just before feeding. To prevent ghrelin degradation, the samples were drawn into tubes maintained at low temperature containing anti-protease 4-(2-aminoethyl) benzenesulfonyl fluoride hydrochloride (AEBSF, Sigma-Aldrich Chemicals) at 2 mg/ml concentration. The samples were all analyzed by a single laboratory in Rotterdam according to the norms and standards described by Beauloye et al. [23]. Total ghrelin (TG), AG and UAG were expressed as pg/ml.

### Other data

Genetic subtypes were separated into deletion and non-deletion (which includes maternal uniparental disomy (mUPD), and more rarely imprinted deficits and translocations). As children with PWS are treated with GH, the age of treatment onset and the dose (mg/kg/day) were recorded. At the time of ghrelin measurement, patients were weighed using an electronic scale and measured in recumbent position if ≤ 3 years old or in supine position if older. BMI was calculated and expressed as z-score, adjusted for age and sex according to the World Health Organization charts. For all patients with EOS, the Cobb angle degree of the spine curve at diagnosis was recorded from the orthopedist’s or radiologist's report. Prescriptions for bracing or surgery were also recorded.

At the same time as the ghrelin measurements, samples were taken for Insulin Like Growth Factor 1 (IGF-1), plasma hemoglobin A1c (HbA1c), and insulin. IGF-1 measurements were performed by automated chemiluminescent immunoassay (IDS-iSYS; Immunodiagnostic Systems), when possible, and expressed in z-score according to sex and age [34]. In patients > 1 year, HbA1c and insulin levels expressed as % and µIU/ml, respectively, were collected. HbA1c was measured using high performance liquid chromatography and insulin was measured using an electro-chemiluminescence competitive binding.

### Statistical analyses

We compared the clinical characteristics of children with PWS with and without EOS using matched analyses (McNemar or Wilcoxon tests depending on the variable). For patients with EOS, age and Cobb angle (or the main angle if double curve) at diagnosis and treatment, if any, were described. We also compared TG, AG, UAG and AG/UAG ratio. Sensitivity analysis was performed, excluding the poorly matched pair. Correlations of Cobb angles with all ghrelin levels were estimated using the Spearman range test. We performed a sensitivity analysis, excluding one patient with a very high Cobb angle outlier (75°). In the longitudinal approach, we compared baseline characteristics and ghrelin levels in the first year of life between infants who developed EOS in the first 4 years of life and those who did not, using Fisher or Mann–Whitney range tests. All analyses were two-sided and performed at the 0.05 significance level. Given their exploratory nature, they were carried out without any adjustment of type I error. STATA v14 software was used.

## Results

The source population was 146 patients with PWS, < 10 years old, and with one or multiple measurements of plasma ghrelin levels. Eighty-three patients who met the eligibility criteria for one or both approaches were selected. Fourteen patients participated in both analyses but with two different ghrelin measurements, one in the first year of life and one later. The procedure for selecting the study population is detailed in Fig. 1. The results of both approaches are presented separately hereafter.

### Case–Control approach

The characteristics of children with EOS and their matched controls (all receiving GH treatment at the time of the measurements) are presented in Table 1. GH treatment onset differed significantly between the two groups, being earlier in the cases.

**Table 1:** Clinical characteristics and TG, AG, UAG and AG/UAG of PWS children with and without EOS

	PWS children with EOS (cases) n = 30	PWS children without EOS (controls) n = 30	*p* value
Sex—girls, n (%)	20 (66.7)	16 (53.3)	0.285
Age—years, median (IQR)	3.7 (2.0–5.2)	3.9 (2.3–5.0)	–
BMI—WHO z-score, median (IQR)	0.0 (− 0.8–1.4)	0.1 (− 0.9–1.4)	–
Genetic subtype—deletion, n (%)	12 (40.0)	19 (63.3)	0.090
Age at start GH—months, median (IQR)	12 (9–13)	13 (10–16)	0.043
GH dose—mg/kg/d, median (IQR)	0.0263 (0.0215–0.0334)	0.0277 (0.0184–0.0321)	0.614
IGF-1—z-score, median (IQR)^a^	1.4 (0.3–2.5)	0.7 (− 0.2–1.7)	0.065
HbA1c—%, median (IQR)^a^	5.4 (5.2–5.6)	5.4 (5.2–5.8)	0.364
Insulin—µUI/ml, median (IQR)^a^	5.2 (2.5–8.2)	4.9 (3.0–7.2)	0.084
TG—pg/ml, median (IQR)	314.0 (207.2–481.0)	317.1 (231.7–618.4)	0.797
AG—pg/ml, median (IQR)	161.3 (89.6–210.6)	194.6 (154.2–256.8)	0.079
UAG—pg/ml, median (IQR)	152.3 (81.7–263.5)	120.1 (71.2–219.2)	0.382
AG/UAG, median (IQR)	0.783 (0.559–1.993)	1.589 (1.166–2.433)	0.005
Age at EOS diagnosis—years, median (IQR)	3.4 (1.8–5.2)		
Cobb angle at diagnosis—degrees, median (IQR)^b^	20 (12–27)		

^a^One scoliotic patient without data on IGF-1, 8 scoliotic patients and 7 non-scoliotic patients without data on HbA1c, and 8 scoliotic patients and 6 non-scoliotic patients without data on insulin

^b^Cobb angle at diagnosis of EOS available only for 27 patients

Table 1 also shows the comparisons of the TG, AG, UAG and AG/UAG between cases and controls. The AG/UAG ratio was significantly lower in children with EOS. No significant differences in TG, AG or UAG were found, despite a trend for lower AG among the cases. In the sensitivity analysis, similar results were obtained (data not shown).

**Fig. 2:**
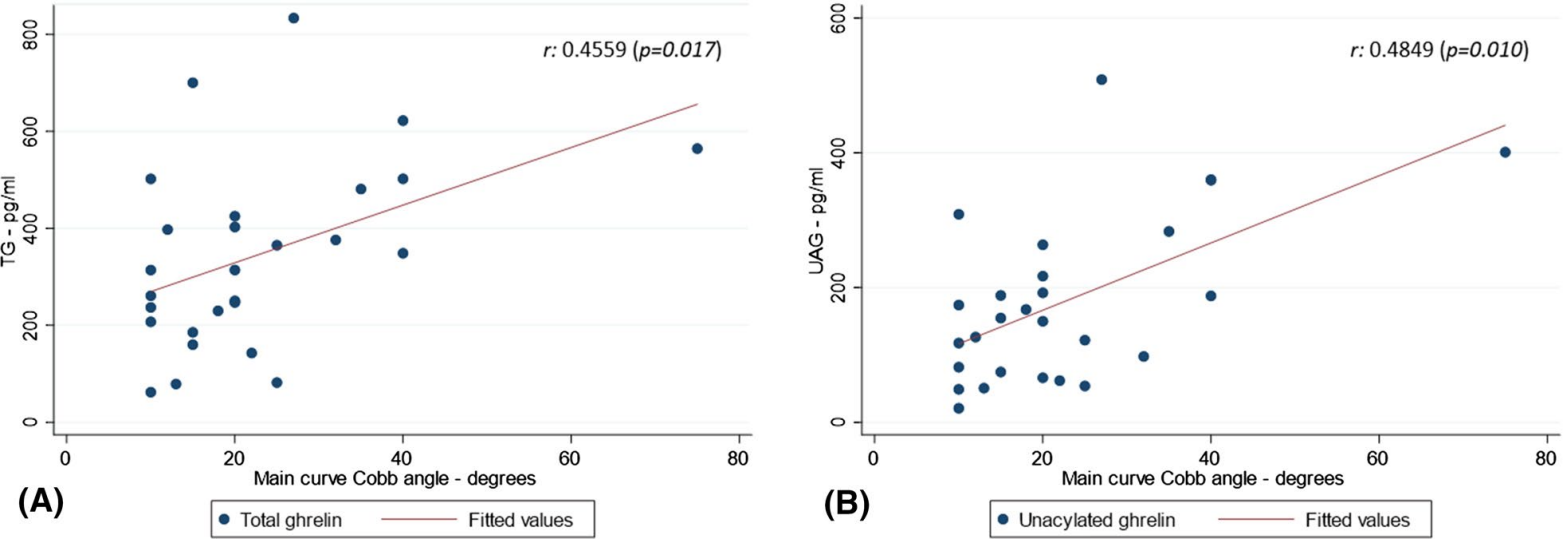
Correlation between TG (A) and UAG (B) with Cobb angle (n = 27)

Of the 30 cases with EOS, nine had a brace prescription at a median age of 2.3 years (interquartile range: IQR, 1.8 to 3.2). No patient had undergone surgical treatment. The Cobb angle value at or near diagnosis was available for 27/30 children with EOS. The TG and UAG levels were positively correlated with the Cobb angle degrees (Fig. 2). No significant correlation was observed between the other ghrelin parameters (AG, AG/UAG) and the Cobb angle, the Spearman correlation coefficients being 0.2284 (*p* = 0.252) and − 0.2623 (*p* = 0.186) respectively. The sensitivity analysis performed by withdrawing the patient who presented an extreme Cobb angle at diagnosis (75°) showed similar results (data not shown).

### Longitudinal approach

Of the 37 infants with a ghrelin measurement in the first year of life (before starting GH treatment), eight (22%) developed EOS during the first 4 years of life. At EOS diagnosis, children were 1.8 years (median, IQR, 1.7 to 1.9), and had a median Cobb angle of 23.5 degrees (IQR, 20 to 35) (missing data for two patients). All patients underwent bracing at a median age of 2.1 years (IQR, 1.7 to 2.4). None underwent surgery during the follow-up period. GH treatment was started at a median age of 11 months (IQR, 10 to 12) in children who developed EOS and a median age of 10 months (IQR, 9 to 12) in those who did not (*p* = 0.420) (missing data for one non-scoliotic patient). The comparison of other clinical characteristics at the time of ghrelin measurement (Table 2) showed no significant difference except a lower IGF-1 z-score in infants who later developed EOS. We also observed a trend of lower BMI z-score in these same infants.

**Table 2:** Clinical and hormonal characteristics of infants with PWS at the time of ghrelin measurement during the first year of life according to their EOS status at the age of 4 years

	PWS infants who later developed EOS n = 8	PWS infants who did not later develop EOS n = 29	*p* value
Sex—girls, n (%)	3 (37.5)	10 (34.5)	1.000
Age—month, median (IQR)	6.1 (3.6–10.3)	7.6 (4.8–9.1)	0.912
BMI—WHO z-score, median (IQR)^a^	− 1.8 (− 2.5– − 1.1)	− 1.1 (− 1.9–0.0)	0.110
Genetic subtype—deletion, n (%)^a^	4 (50.0)	12 (42.9)	1.000
IGF-1—z-score, median (IQR)^b^	− 2.1 (− 2.5– − 1.3)	− 1.1 (− 1.7– − 0.4)	0.008

^a^One non-scoliotic patient without data on height and unspecified genetic subtype

^b^Two patients, one in each group, without data on IGF-1

**Fig. 3:**
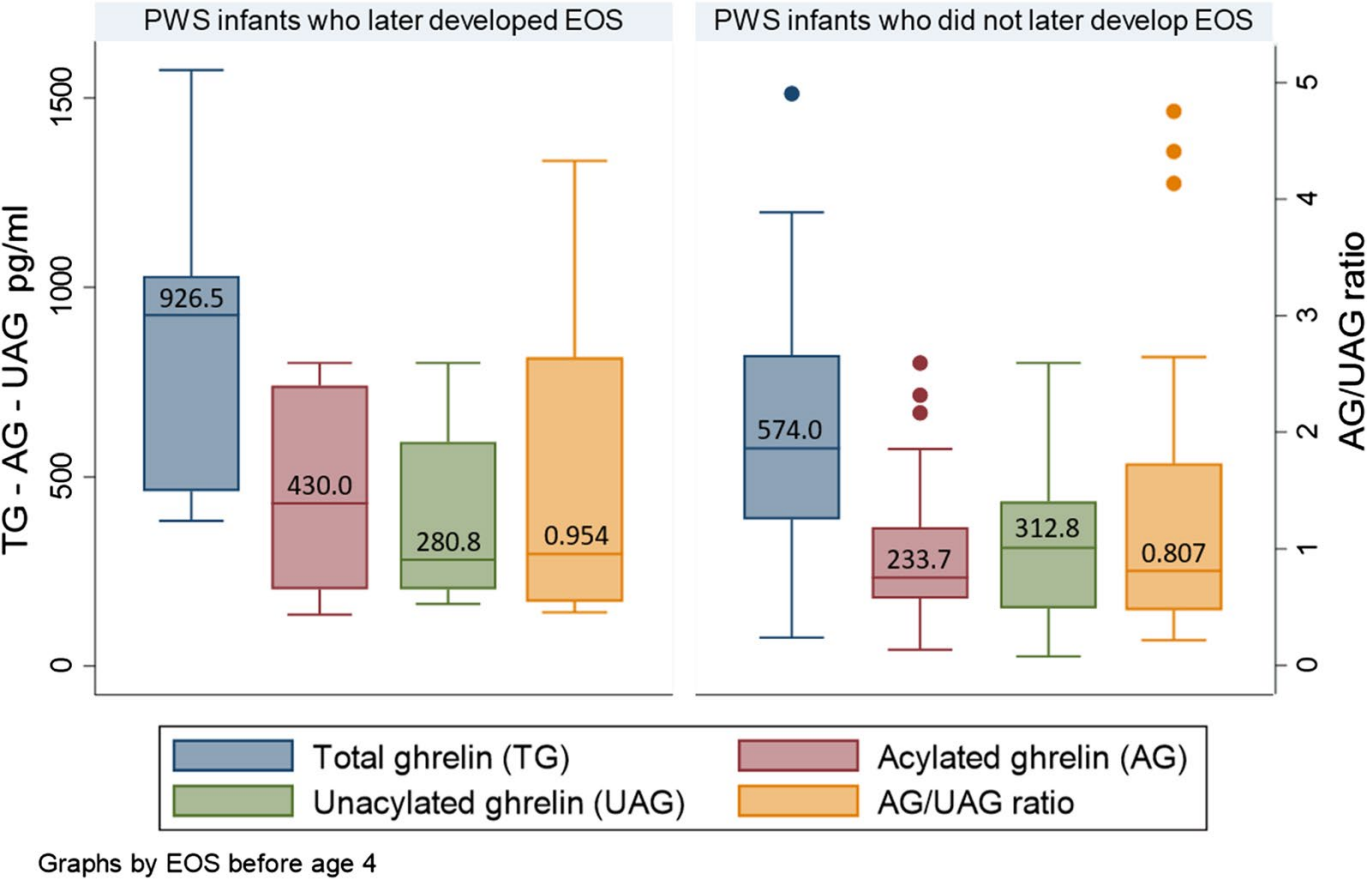
Comparison of TG, AG, UAG and AG/UAG among infants with PWS at the first year of life according to their EOS status at the age of 4 years

Figure 3 shows the distribution of ghrelin parameters according to EOS status at the age of 4. The median levels of TG and AG were almost two times higher in the group that later developed EOS although the difference did not reach statistically significance, respectively 926.5 pg/ml vs 574.0, *p* = 0.140*, *and 430.0 pg/ml vs 233.7, *p* = 0.135. UAG and AG/UAG did not significantly differ between the two groups (*p* = 0.483 and 0.555).

## Discussion

### Key results

Our study shows that children with PWS have a significantly lower AG/UAG ratio at the time of their EOS diagnosis than age- and BMI-matched PWS children without scoliosis, with a tendency for lower AG, suggesting that ghrelin may be a biomarker of scoliosis in PWS. The Cobb angle in the EOS children positively correlated with TG and UAG, suggesting that higher TG and UAG are linked to scoliosis severity. Moreover, a trend for high TG and AG levels were observed in the first year of life in patients who later developed EOS compared to those who did not. Although the difference did not reach statistical significance perhaps due to lack of power, it could be hypothesized that TG and AG levels may be associated with the future occurrence of EOS. This hypothesis should be verified in a larger sample.

### Strengths and limitations

To our knowledge, this is the first in-depth study to investigate the role of ghrelin isoforms in EOS in children with PWS. PWS may be used as a model for a broader understanding of the pathophysiology of scoliosis, and particularly the role of hyperghrelinemia.

We used a pediatrics population-based database in which data are recorded from pediatric patients followed by the FRC-PWS. In France, PWS patients are followed closely in one of the 20 hospitals designated as competence centers and are evaluated once a year by one of the two pediatric sites of the FRC-PWS (Toulouse and Paris). During visits to the FRC-PWS, patients are seen by an orthopedist and usually undergo an X-ray. These experts make or confirm the scoliosis diagnosis. Information on care, treatment, and biological measurements is updated during patient follow-up and included in a systematic and standardized manner in the FRC-PWS database. For these reasons, we are confident that our data are comprehensive.

The sample selection resulted from several limitations, particularly the availability of ghrelin measurements only collected as part of clinical research. Measurements of the ghrelin isoforms have been done with accuracy, samples were extracted at only two centers using strict and standardized sampling and collection procedures, and were centralized in a single laboratory.

We used two complementary approaches to explore pre-specified hypotheses. No prior power calculations were thus made for this exploratory study, not designed to explore causal effects. The main possible confounding factors were taken into consideration when selecting the study population. In both approaches, we homogenized the samples by age and GH treatment. In the case–control approach, we also matched by BMI.

### Interpretation

Several studies have documented the functions of ghrelin on bone cells [6, 8]. A recent study showed that chondrocytes of AIS patients express more ghrelin receptors and higher levels of expression of specific genes that can lead to impaired cartilage development [14]. Asymmetric vertebral growth has been implicated as a possible etiologic factor in AIS pathogenesis [12]. Longitudinal growth of the vertebral bodies in patients with AIS seems disproportionate and faster than in age- and sex-matched controls and takes place mainly by endochondral ossification [28, 35]. In PWS, no publication has yet evaluated whether alterations in vertebral growth occur in either EOS or later-onset scoliosis.

In addition to high ghrelin levels, patients with AIS have abnormal bone quality, which may contribute to the pathophysiology of scoliosis [36]. Numerous articles have investigated the relationship between ghrelin and bone mineral density (BMD) with inconsistent results. Xiao et al. suggested a dysregulation of ghrelin-regulated pathways that decreases the osteogenic ability of osteoblasts in patients with AIS osteopenia, although they only measured total ghrelin [15]. Although little is known, there may be differential roles between AG and UAG in BMD. A recent study demonstrated a correlation between UAG and the decline in BMD after gastric bypass in obese patients [37].

Indeed, ghrelin secretion may interact with GH/IGF1 axis. In our case–control approach, children with EOS tended to have higher IGF-1 levels on GH treatment compared to children without EOS. This might indicate higher sensitivity to GH treatment in the case group. A recent study suggested that PWS patients with mUPD have increased GH sensitivity [38] and we also found a higher proportion of patients with mUPD in the group with EOS that was also reported in the literature [30]. In the longitudinal approach, while IGF-1 levels were low for all infants, they were even lower for those who later developed scoliosis. These lower IGF-1 levels may be related to the BMI z-scores we observed in infants who later developed EOS that tended to be lower. In de Lind van Wijngaarden's study [31], lower BMI was observed in children with PWS with EOS compared with the older children with AIS-like scoliosis.

Nevertheless, no study has yet confirmed an association between GH sensitivity and scoliosis development in the PWS population. It was, however, demonstrated that GH treatment does not induce scoliosis [26], and worsening scoliosis is likely related to the growth spurt. Furthermore, little is known about the association of ghrelin and GH sensitivity, although negative correlations between IGF-1 levels and AG and UAG have been observed in children with PWS [24]. Further studies are needed to explain the underlying hormonal interactions in PWS and their impact on scoliosis development.

## Conclusion

Our results suggest that ghrelin may play a role in the pathophysiology of scoliosis in patients with PWS. Currently, at the FRC-PWS, we are implementing a prospective follow-up of a larger sample of patients with PWS undergoing ghrelin measurement who will be evaluated for scoliosis in a standardized manner to demonstrate if ghrelin could be used as a biomarker for EOS. If our findings are confirmed, ghrelin levels during the first year of life particularly in those infants with mUPD and low IGF-1 values could predict the development and progression of the spinal deformity. It would be interesting to study whether other rare syndromes with EOS also present ghrelin system abnormalities. If ghrelin is an important factor of vulnerability of EOS, new therapeutic approaches with drugs targeting the ghrelin system may be an interesting opportunity for treating and/or preventing scoliosis pharmacologically.

## Data Availability

The datasets generated and analyzed during the current study are not publicly available due to medical privacy but are available from the corresponding author on reasonable request.

## Abbreviations

PWS: Prader–Willi syndrome
EOS: Early-onset scoliosis
BMI: Body mass index
TG: Total ghrelin
AG: Acylated ghrelin
UAG: Unacylated ghrelin
GH: Growth hormone
AIS: Adolescent idiopathic scoliosis
FRC-PWS: French Reference Center for PWS
IGF-1: Insulin Like Growth Factor 1
mUPD: Maternal uniparental disomy
HbA1c: Hemoglobin A1c
IQR: Interquartile range
BMD: Bone mineral density
